# Supplementary material for: Splice-Junction-Based Mapping of Alternative Isoforms in the Human Proteome
Source: Cell Rep. Author manuscript; Available in PMC 2020 Jan 15. (PMC6961840; doi:10.1016/j.celrep.2019.11.026)
Supplement: 2 [file NIHMS1546469-supplement-2.zip › DF1/PXD010154/Lung/Lung_31_NDUFA10_LLQYSDALEHLLTTGAVFLGSATSASAR.pdf]

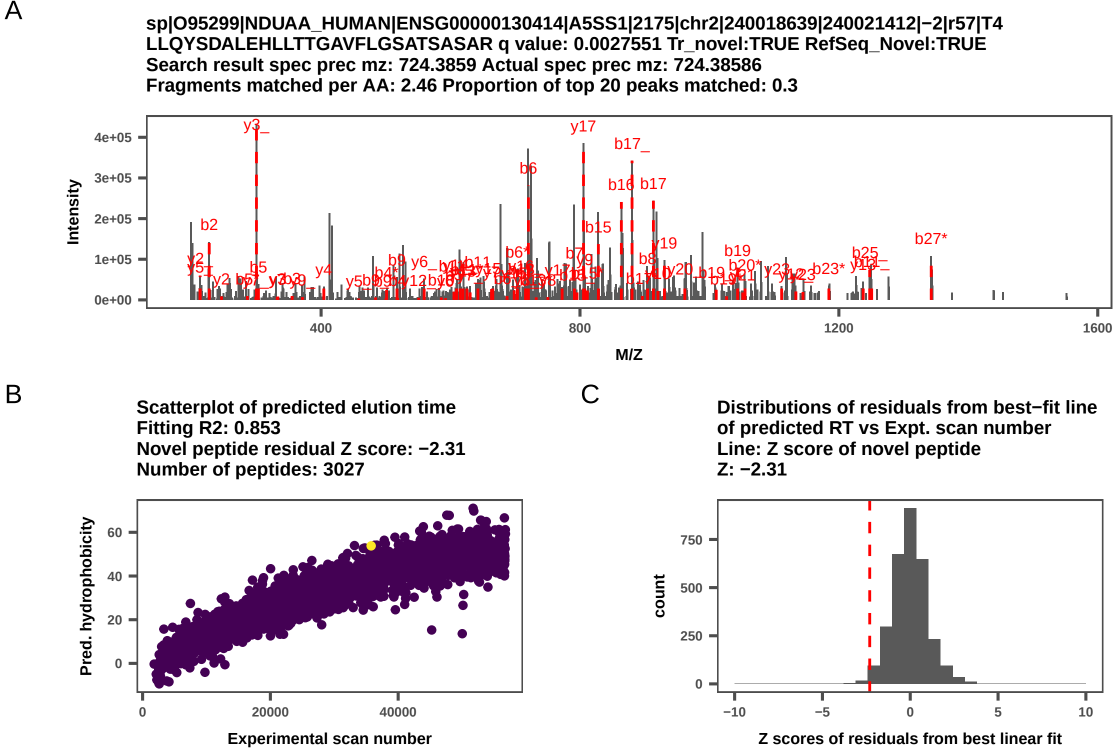
A
sp|O95299|NDUAA_HUMAN|ENSG00000130414|A5SS1|2175|chr2|240018639|240021412|−2|r57|T4
LLQYSDALEHLLTTGAVFLGSATSASAR q value: 0.0027551 Tr_novel:TRUE RefSeq_Novel:TRUE
Search result spec prec mz: 724.3859 Actual spec prec mz: 724.38586
Fragments matched per AA: 2.46 Proportion of top 20 peaks matched: 0.3
Intensity
4e+05
3e+05
2e+05
1e+05
0e+00
400
800
1200
1600
M/Z
y3_
y17
b17_
b6
b16
b17
b2
b15
y19
b19
b27*
b25
B
Scatterplot of predicted elution time
Fitting R2: 0.853
Novel peptide residual Z score: −2.31
Number of peptides: 3027
Pred. hydrophobicity
60
40
20
0
0
20000
40000
Experimental scan number
C
Distributions of residuals from best−fit line
of predicted RT vs Expt. scan number
Line: Z score of novel peptide
Z: −2.31
count
750
500
250
0
−10
−5
0
5
10
Z scores of residuals from best linear fit
